# Dataset on the generation and inhibitor-based selection of *Candida utilis* mutants for enhanced protein production

**DOI:** 10.1016/j.dib.2026.113177

**Published:** 2026-08-26

**Authors:** Jelizaveta Palcevska, Zane Kusnere, Svetlana Raita, Zane Geiba, Ilze Vamza

**Affiliations:** Riga Technical University, Institute of Energy Systems and Environment, Azenes street 12/1, LV 1048, Riga, Latvia

**Keywords:** Amino acid inhibitors, *Candida utilis*, Mutant screening, Mutagenesis, Protein-rich biomass, Response surface methodology, Single-cell protein

## Abstract

This data article presents a dataset on the generation and inhibitor-based selection of *Candida utilis* mutants for enhanced protein production. The experimental approach combines random mutagenesis using ethyl methanesulfonate with screening in media supplemented with amino acid biosynthesis inhibitors. These inhibitors impose selective pressure on metabolic pathways related to amino acid synthesis.

The dataset includes experimental data from medium screening, mutagenesis, inhibitor-based mutant selection, and cultivation medium optimization using Response Surface Methodology. The applied screening concept uses inhibitory compounds to identify mutant cells that can grow under conditions where the wild-type strain is suppressed. Measured parameters include biomass concentration, protein content, protein yield, optical density, survival rates, and amino acid composition. The dataset also provides replicate measurements, mean values, and standard deviations.

The dataset documents the complete workflow from medium screening to mutant selection and process optimization, including validation under shake-flask and bioreactor conditions. It identifies an optimized workflow for *C. utilis* strain improvement for single-cell protein production. Among the generated mutants, GA0.4/39–2 showed the best overall performance and was successfully validated in 5 L bioreactor cultivation, reaching a maximum biomass concentration of 23.87 ± 0.75 g/L and a maximum protein yield of 11.49 g/L. The dataset provides a reusable framework for microbial strain improvement, single-cell protein production, and bioprocess optimization that may also support the development of similar workflows for other microorganisms.

Specifications Table

**Table:** 

Subject	Biology
Specific subject area	Biotechnology; Microbial protein production; fermentation process optimization; strain improvement
Type of data	Tables; Graphs; Figures Raw; Processed; Analyzed
Data collection	Data were obtained from shake-flask and bioreactor experiments with *Candida utilis,* including medium screening, optimization, EMS mutagenesis, inhibitor-based mutant screening and Response Surface Methodology. Biomass concentration was determined primarily by gravimetric analysis (dry cell weight), while optical density (OD600) was used in microplate experiments during inhibitor screening. Protein content was measured using the BCA assay, and amino acid composition was analyzed by HPLC. All experiments were performed in triplicate unless otherwise stated. The dataset includes raw data, mean values, and standard deviations.
Data source location	Data were collected at Riga Technical University, Institute of Energy Systems and Environment, Azenes street 12/1, LV 1048, Riga, Latvia, in Biosystem laboratory
Data accessibility	Data are available at Repository name: Zenodo Data identification number: https://doi.org/10.5281/zenodo.19854128 Direct URL to data: https://zenodo.org/records/19854128
Related research article	None

## 1 Value of the Data

• This dataset provides a comprehensive workflow for generating and screening *Candida utilis* mutants using EMS mutagenesis, amino acid biosynthesis inhibitors, and microplate-based selection, which can serve as a reference for developing similar strain improvement strategies in other microorganisms.
• The dataset includes key measured parameters such as biomass concentration, protein content, protein yield, optical density, survival rates, and amino acid composition, together with replicate measurements and standard deviations, enabling comparative analyses and independent validation of experimental results.
• The dataset integrates all major stages of the experimental workflow, including medium screening, mutagenesis, inhibitor-based selection, Response Surface Methodology optimization, and validation in shake-flask and bioreactor systems, allowing researchers to reproduce and adapt the complete strain development workflow.
• The availability of raw and processed data, including experimental design matrices and Response Surface Methodology datasets, supports data reuse, mathematical modelling, and validation of optimization approaches.
• These data provide a reusable resource for researchers working in microbial biotechnology to design, reproduce, compare, and adapt similar strain improvement and bioprocess optimization strategies for single-cell protein production using different microorganisms and cultivation substrates.

## 2 Background

The increasing demand for alternative protein sources has led to growing interest in single-cell protein (SCP) produced by microorganisms [1]. The protein content and essential amino acid (EAA) composition of microbial biomass are largely genetically determined but can also be influenced by cultivation conditions. Improvements in SCP production are commonly achieved through two approaches: optimization of fermentation processes [[2], [3], [4]] and modification of strain properties through mutagenesis [5]. *Candida utilis* is widely studied as an SCP-producing yeast because it can efficiently utilize diverse agro-industrial by-products and exhibits high biomass and protein productivity under controlled cultivation conditions [6,7]. These characteristics make it an attractive candidate for strain improvement strategies aimed at increasing biomass production and protein quality.

Random mutagenesis using ethyl methanesulfonate (EMS) is widely used to generate genetic variability [8,9]. Selection of mutants can be supported by amino acid biosynthesis inhibitors, which impose selective pressure on metabolic pathways involved in nitrogen assimilation and amino acid synthesis. This principle has been applied in the selection of microorganisms with enhanced production of metabolites such as fatty acids and carotenoids, where inhibition of biosynthetic pathways enables identification of overproducing strains [10,11]. However, its application for selecting protein-overproducing mutants remains largely unexplored. In this work, amino acid biosynthesis inhibitors - including glufosinate-ammonium (GA), S-(2-aminoethyl)-l-cysteine hydrochloride (AEC), and l-methionine sulfoximine (MSO) - were used as selective agents following EMS mutagenesis. Microplate-based screening was applied to evaluate growth responses under selective conditions, while medium composition was investigated using shake-flask experiments and optimized using Response Surface Methodology (RSM).

The resulting dataset provides a comprehensive resource covering mutant generation, inhibitor-based selection, cultivation medium optimization, and validation experiments for *C. utilis*. By integrating raw and processed data from all experimental stages, it provides a valuable resource for researchers interested in microbial strain improvement, SCP production, comparative studies, and the development of new protein-producing microbial strains. Unlike datasets focusing on a single experimental stage, this dataset integrates all experimental stages within a single reproducible framework. The dataset also expands the available knowledge on the use of amino acid biosynthesis inhibitors as selective tools for SCP strain improvement.

## 3 Data Description

This article presents a dataset supporting the generation, screening, and evaluation of protein-producing mutants of *C. utilis*, as well as the optimization of cultivation conditions. The dataset is provided as a spreadsheet (.xlsx) containing multiple worksheets, each corresponding to a specific experimental stage [12].

Worksheets 1 and 2 (entitled “Media test Nr1” and “Media test Nr2”) present data from two sequential shake-flask medium screening experiments. These sheets include medium composition (g/L), biomass concentration (g/L), protein content (%), protein yield (g/L), individual replicate measurements, mean values, and standard deviations. Representative biomass data from the second medium screening experiment are presented in Table 1.

**Table 1:** *C. utilis* growth comparison in different molasses-rich media (mean ± standard deviation, n = 3).

Media No.	Dry cell weight, g/L				
	72 h	96 h	120 h	144 h	168 h
1	13.63 ± 0.29	13.23 ± 0.25	11.87 ± 0.21	11.90 ± 0.17	12.30 ± 0.10
2	13.83 ± 0.42	13.63 ± 0.21	12.20 ± 0.10	12.53 ± 0.15	12.43 ± 0.06
3	14.60 ± 0.20	13.40 ± 0.10	12.37 ± 0.25	12.50 ± 0.17	12.67 ± 0.15
4	13.30 ± 0.36	15.47 ± 0.21	13.70 ± 0.10	13.93 ± 0.23	13.90 ± 0.46
5	13.83 ± 0.25	15.73 ± 0.23	14.15 ± 0.26	13.90 ± 0.31	14.25 ± 0.26
6	14.33 ± 0.06	15.90 ± 0.10	13.93 ± 0.15	13.80 ± 0.26	14.23 ± 0.32
7	12.90 ± 0.26	14.97 ± 0.38	15.53 ± 0.31	14.97 ± 0.06	15.10 ± 0.10
8	13.97 ± 0.32	16.93 ± 0.15	15.63 ± 0.15	15.07 ± 0.06	15.00 ± 0.17
9	14.53 ± 0.21	17.17 ± 0.23	15.57 ± 0.15	15.03 ± 0.29	15.43 ± 0.15
10	12.27 ± 0.29	14.97 ± 0.06	17.07 ± 0.40	16.43 ± 0.21	16.43 ± 0.06
11	13.80 ± 0.61	16.07 ± 0.06	17.67 ± 0.21	17.17 ± 0.25	17.27 ± 0.15
12	14.07 ± 0.15	16.93 ± 0.15	17.97 ± 0.06	17.10 ± 0.26	17.40 ± 0.10

In the first screening experiment (“Media test Nr1”), four media containing different carbon and nitrogen sources were compared. The nutrient-rich glycerol-based control medium supported the highest biomass concentration, reaching 35.10 ± 1.15 g/L after 168 h, and the highest protein yield of 9.52 g/L. Among the media containing ammonium sulfate as an inorganic nitrogen source, the molasses-based medium showed the best performance, reaching 21.30 ± 0.20 g/L biomass, 34.18% protein content, and a protein yield of 7.28 g/L after 168 h. In comparison, the glucose- and glycerol-based media containing ammonium sulfate produced biomass concentrations of 6.43 ± 0.35 and 4.57 ± 0.21 g/L, respectively. These data supported the use of molasses as the carbon source in the subsequent medium screening experiment.

In the second screening experiment (“Media test Nr2”), molasses concentrations of 80–112 g/L and C/N ratios of 10–20 were evaluated at a constant pH of 5.0. Media containing 100–112 g/L molasses generally supported higher biomass production than those containing 80–90 g/L molasses. The highest biomass concentration, 17.97 ± 0.06 g/L, was obtained with Medium 12 containing 112 g/L molasses at a C/N ratio of 20 after 120 h. Medium 9, containing 100 g/L molasses at a C/N ratio of 20, reached 17.17 ± 0.23 g/L after 96 h and was selected for subsequent mutant screening because it provided a balance between high biomass production, lower substrate consumption, and a shorter cultivation period compared with the 112 g/L molasses formulations.

**Fig. 1:**
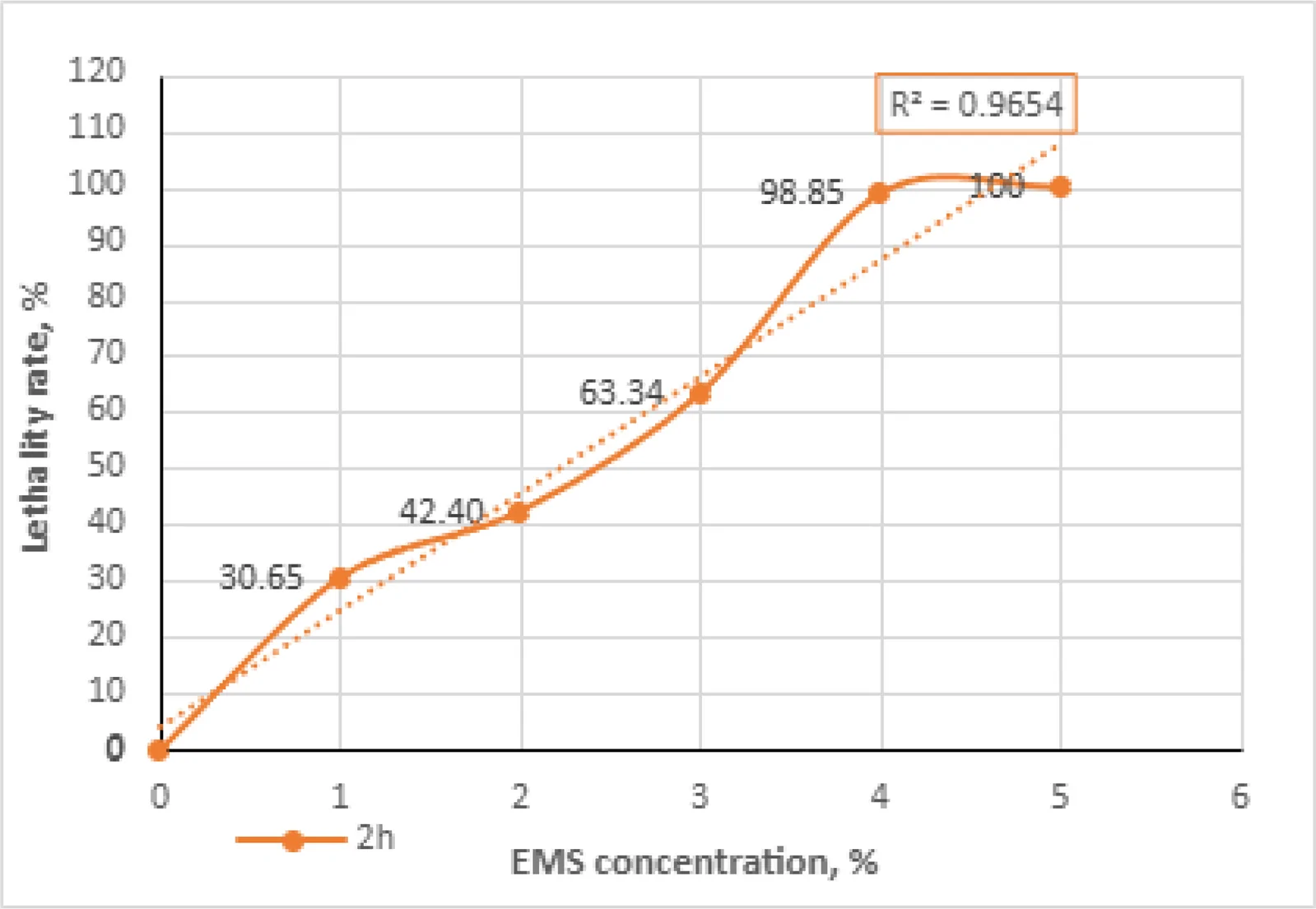
*C. utilis* lethality rate depending on EMS concentration.

Worksheet 3 (entitled “Lethality rate on EMS”) contains data from mutagenesis experiments. The data include EMS concentration, colony-forming units (CFU), and calculated survival and mortality rates (%). Representative lethality data are presented in Fig. 1.

The dataset demonstrates a clear concentration-dependent increase in EMS lethality, with cell inhibition increasing from 30.65% at 1% EMS to complete lethality at 5% EMS. A concentration of 4% EMS resulted in 98.85% lethality and was selected for subsequent mutant generation because it provided near-complete lethality while still allowing recovery of a small proportion of surviving cells.

Worksheet 4 (entitled “Effect of AA inhibitors”) presents growth data obtained in microplate experiments at different concentrations of amino acid biosynthesis inhibitors. The dataset includes inhibitor type and concentration, optical density (OD600) measurements, and growth responses relative to control conditions. A representative dataset illustrating the effect of glufosinate-ammonium is presented in Table 2.

**Table 2:** Effect of GA concentration on *C. utilis* cell concentration and optical density.

GA conc., M	Cell concentration, cells/mL	Optical Density
0	2.17E+08	1.733
0.2	1.87E+07	0.091
0.25	4.88E+06	0.015
0.3	4.94E+06	0.007
0.35	5.06E+06	0.028

The dataset demonstrates distinct inhibitory effects of the tested amino acid biosynthesis inhibitors on *C. utilis* growth. AEC showed only a weak inhibitory effect, whereas MSO caused moderate growth inhibition at higher concentrations. In contrast, GA was the most effective inhibitor, producing complete growth inhibition from 0.1 M onwards. Based on the additional inhibition experiments, 0.4 M GA was selected as the selective concentration for subsequent mutant screening experiments.

Worksheet 5 (entitled “Mutant test”) includes shake-flask data for the obtained mutants (see Table 3). The dataset provides biomass concentration (g/L), protein content (%), protein yield (g/L), and amino acid composition determined in both molasses-based (MAM) and glycerol-peptone (GPM) media.

**Table 3:** Obtained mutant and wild-type strain growth comparison in different media (mean ± SD, n = 3).

Strain	Biomass indicators at 168 h of cultivation in MAM medium			Biomass indicators at 168 h of cultivation in GPM medium		
	DCW, g/L	Protein, %	Protein yield, g/L	DCW, g/L	Protein, %	Protein yield, g/L
WT	15.43 ± 0.15	41.00 ± 1.34	6.33 ± 0.14	35.10 ± 1.15	27.11 ± 1.60	9.52 ± 0.59
GA0.4/8–2	16.07 ± 0.60	44.71 ± 4.12	7.18 ± 0.67	27.67 ± 1.23	37.2 ± 3.01	10.29 ± 1.12
GA0.4/9–2	15.17 ± 0.50	–	–	24.23 ± 0.12	–	–
GA0.4/17–2	15.97 ± 0.06	40.98 ± 1.28	6.54 ± 0.23	29.10 ± 0.26	37.69 ± 0.87	10.97 ± 0.25
GA0.4/22–2	14.73 ± 0.25	–	–	25.10 ± 0.44	–	–
GA0.4/24–2	16.00 ± 0.56	44.49 ± 2.40	7.12 ± 0.63	27.93 ± 2.08	35.96 ± 1.39	10.04 ± 1.01
GA0.4/29–2	14.33 ± 0.35	43.16 ± 0.86	6.19 ± 0.07	27.90 ± 0.72	36.00 ± 0.74	10.04 ± 0.31
GA0.4/31–2	23.63 ± 0.31	–	–	19.23 ± 0.60	–	–
GA0.4/39–2	23.67 ± 0.15	43.2 ± 1.41	10.22 ± 0.28	28.10 ± 0.89	38.30 ± 1.00	10.76 ± 0.10
GA0.4/41–2	15.73 ± 0.75	–	–	19.83 ± 0.75	–	–

The dataset enables comparison of mutant performance with the wild-type strain under two cultivation conditions. In MAM medium, mutant GA0.4/39–2 showed the highest biomass concentration (23.67 ± 0.15 g/L) and protein yield (10.22 ± 0.28 g/L), substantially exceeding the wild-type strain. In GPM medium, several mutants achieved higher protein yields than the wild type, with the highest value observed for GA0.4/17–2 (10.97 ± 0.25 g/L), while GA0.4/39–2 showed the highest protein content (38.30 ± 1.00%). The dataset also includes amino acid profiles of the selected mutants cultivated in both media, showing amino acid compositions generally comparable to or improved relative to the wild-type strain, including increased lysine content in several mutants, particularly GA0.4/17–2 and GA0.4/39–2. These results identified GA0.4/39–2 as the most promising mutant for further cultivation studies.

**Fig. 2:**
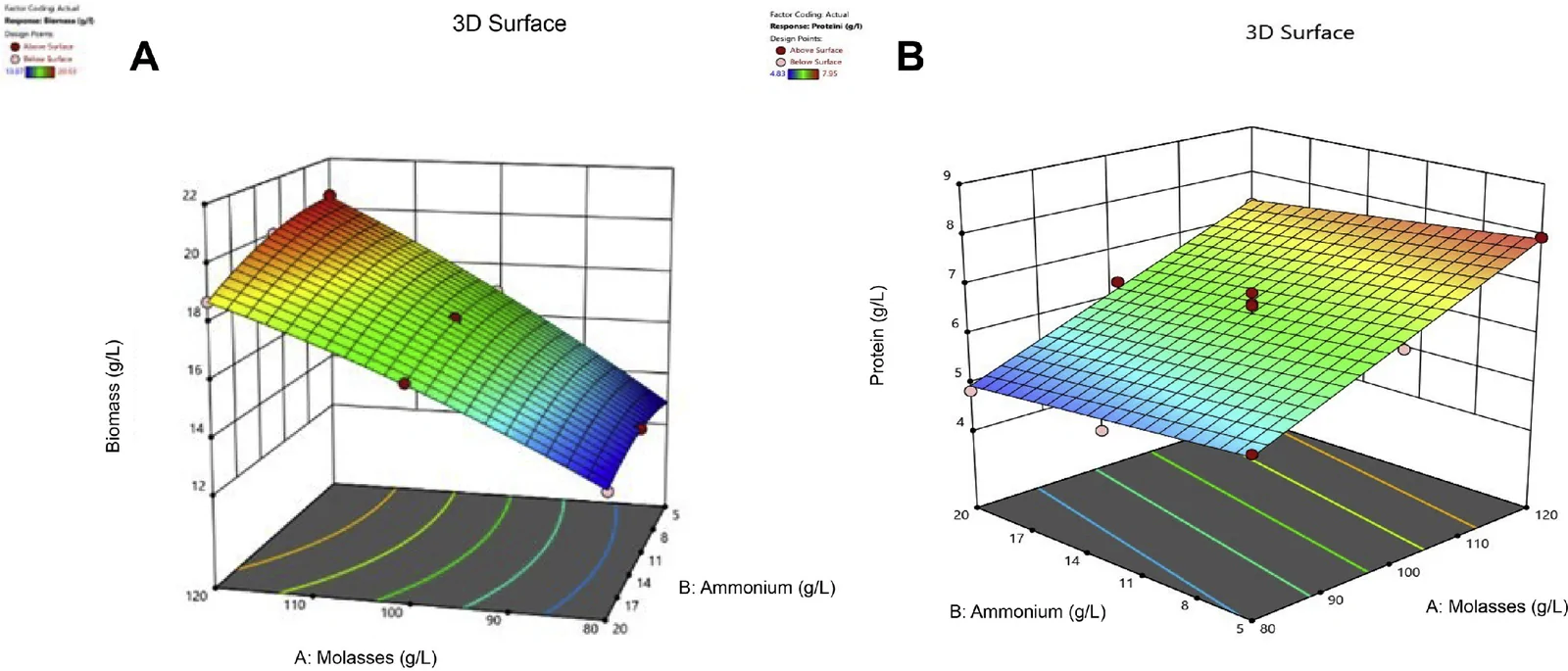
3D response surface plots demonstrated the influence of the interaction between the two independent variables on biomass yield (A) and protein yield (B) in the initial RSM experiment.

**Fig. 3:**
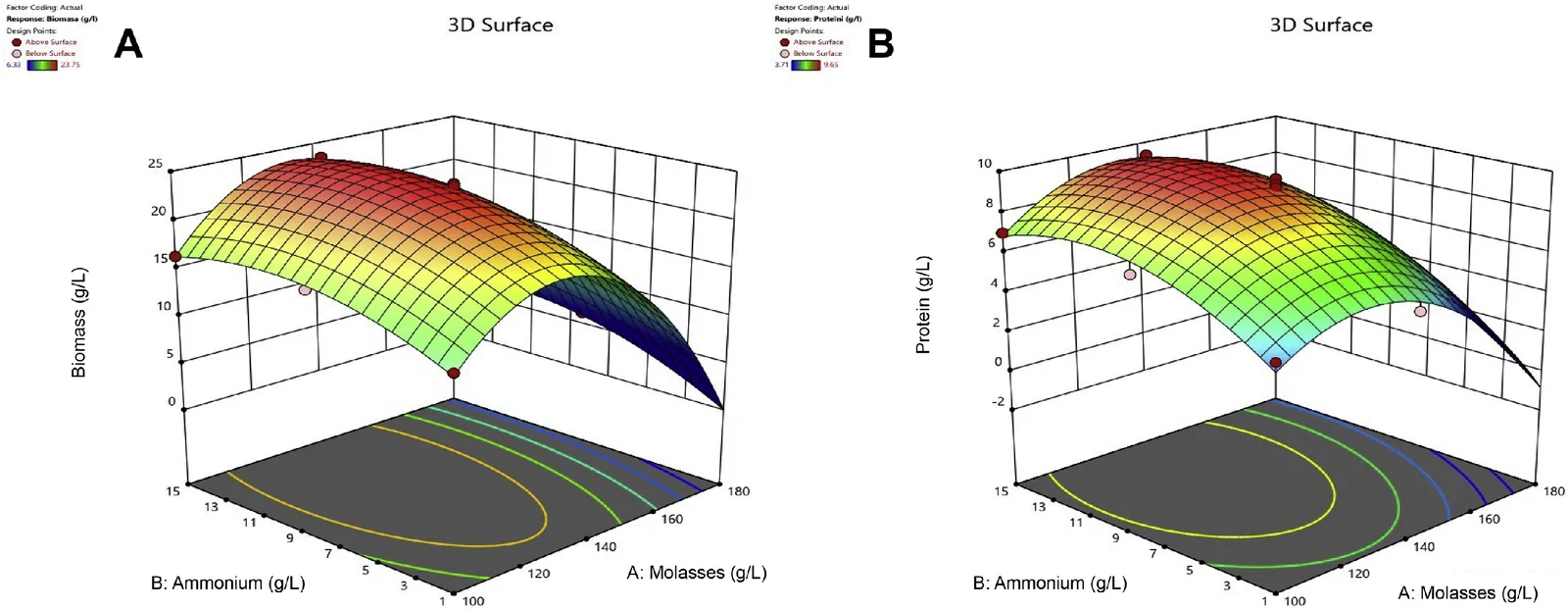
3D response surface plots demonstrated the influence of the interaction between the two independent variables on biomass yield (A) and protein yield (B) in the expanded RSM experiment.

Worksheets 6, 7, and 8 (entitled “RSM Nr1”, “RSM Nr2”, and “RSM Validation”) contain experimental design matrices and results from RSM optimization. These sheets include coded and actual factor levels (molasses and (NH_4_)_2_SO_4_ concentrations), measured biomass and protein yields, and model-predicted responses. Fig. 2, Fig. 3 represent two sequential RSM experiments: the first within the initial factor range and the second within an expanded range to capture the optimal region.

The first RSM experiment showed that increasing molasses concentration enhanced both biomass and protein production within the tested range, with the highest responses obtained at 120 g/L molasses and 5 g/L (NH_4_)_2_SO_4_. Because the predicted optimum was located at the boundary of the design space, a second RSM experiment was performed using an expanded factor range. The second RSM identified a well-defined optimum region at intermediate substrate concentrations, with the highest biomass (23.75 ± 0.65 g/L) and protein yield (9.65 ± 0.34 g/L) obtained at 140 g/L molasses and 8 g/L (NH_4_)_2_SO_4_. Model optimization predicted an optimum medium containing 121.0 g/L molasses and 11.2 g/L (NH_4_)_2_SO_4_, which was subsequently evaluated in the RSM validation experiment. The validation experiment confirmed the suitability of the optimized medium, with maximum biomass reached on the fifth day of cultivation; the experimental biomass values were slightly lower than those predicted by the model.

Worksheet 9 (entitled “Antifoam test”) contains shake-flask data comparing three antifoam agents (J673A, SB2121, and Antifoam C) at different concentrations (0.1–1.0%) during cultivation of *C. utilis* mutant GA0.4/39–2. The dataset includes biomass concentration (g/L) measured over time with corresponding standard deviations. The results showed that Antifoam C maintained biomass production comparable to the control at all tested concentrations, whereas J673A caused moderate growth inhibition and SB2121 strongly reduced biomass production. Based on these results, Antifoam C 0.5% was selected for subsequent bioreactor experiments. Representative biomass data are presented in Table 4.

**Table 4:** *C. utilis* mutant GA0.4/39–2 growth comparison in media with different antifoam agents (mean ± SD, n = 3).

	Dry cell weight, g/L				
	72 h	96 h	120 h	144 h	168 h
Without antifoam	13.40 ± 0.00	18.47 ± 0.25	20.20 ± 0.17	18.33 ± 0.25	18.23 ± 0.12
J673A 0.1%	15.37 ± 0.42	18.67 ± 0.15	16.60 ± 0.10	15.53 ± 0.25	15.53 ± 0.12
J673A 0.5%	13.80 ± 0.30	15.87 ± 0.25	14.27 ± 0.15	13.33 ± 0.40	13.33 ± 0.51
J673A 1%	16.17 ± 1.79	18.33 ± 0.47	17.57 ± 0.40	16.67 ± 0.31	16.20 ± 0.52
SB2121 0.1%	10.20 ± 0.36	12.10 ± 0.62	11.20 ± 0.84	10.35 ± 0.68	11.00 ± 0.67
SB2121 0.5%	6.93 ± 1.10	9.40 ± 0.87	8.70 ± 0.30	8.20 ± 0.10	8.50 ± 0.20
SB2121 1%	5.70 ± 0.17	7.07 ± 0.21	9.77 ± 0.64	9.20 ± 0.69	8.93 ± 0.47
Antifoam C 0.1%	13.90 ± 0.20	18.33 ± 0.15	20.10 ± 0.36	18.23 ± 0.15	18.13 ± 0.46
Antifoam C 0.5%	13.97 ± 0.21	18.60 ± 0.17	20.10 ± 0.36	18.10 ± 0.40	18.13 ± 0.06
Antifoam C 1%	13.93 ± 0.21	18.20 ± 0.53	19.97 ± 0.31	18.13 ± 0.06	18.47 ± 0.12

Worksheet 10 (entitled “Bioreactor 5 L”) includes time-course data from controlled 5 L bioreactor cultivation of *C. utilis* mutant GA0.4/39–2 under optimized cultivation conditions. The dataset contains biomass concentration (g/L), protein content (%) and/or protein yield (g/L), as well as process parameters such as pH, dissolved oxygen, and temperature. The data demonstrate the fermentation kinetics, showing maximum biomass production (23.87 g/L) after 25 h of cultivation, while cumulative protein productivity reached its maximum (0.460 g/L/h) after 25 h, identifying the optimal harvesting window depending on the target response. The dataset also includes sucrose consumption data, demonstrating complete substrate utilization within the first 16 h of cultivation. Representative fermentation kinetics are presented in Table 5.

**Table 5:** Time-course data of biomass and protein production during bioreactor cultivation of *C. utilis* GA0.4/39–2 (mean ± SD, n = 2).

Time (h)	Biomass (g/L)	Cell concentration (cells/mL)	Protein content (%)	Protein yield (g/L)	Protein productivity (g/L/h)
0	3.13 ± 0.57	3.60E+08	-	-	-
16	13.80±0.92	7.39E+08	45.30 ± 1.13	6.25	0.391
25	23.87±0.75	1.54E+09	48.15 ± 0.35	11.49	0.460
40	20.87±0.49	1.66E+09	50.45 ± 1.20	10.53	0.263
51	20.70±0.85	1.97E+09	49.20 ± 1.70	10.18	0.199
69	19.97±0.95	2.18E+09	52.20 ± 1.56	10.42	0.151

Across all worksheets, units and experimental conditions are specified within each sheet. Overall, the dataset provides a reusable resource for developing, reproducing, and optimizing microbial strain improvement workflows and cultivation strategies for single-cell protein production. The dataset identifies key experimental conditions for *C. utilis* strain improvement and SCP production. Among the generated mutants, GA0.4/39–2 consistently showed the best overall performance across different cultivation media. Its performance was successfully validated in a 5 L bioreactor, where it reached a maximum biomass concentration of 23.87 ± 0.75 g/L and a maximum protein yield of 11.49 g/L.

## 4 Experimental Design, Materials and Methods

### 4.1 Microorganism and cultivation conditions

The yeast *Candida utilis* LMKK 44 was used. Stock cultures were maintained on malt extract agar at 4 °C. Inoculum was prepared in liquid medium and cultivated at 28 °C and 250 rpm to obtain ∼1 × 10⁶ cells/mL. Shake-flask experiments were performed in 250 mL Erlenmeyer flasks with a working volume of 50 mL at 30 °C and 250 rpm.

### 4.2 Medium screening and analytical methods

Different carbon and nitrogen sources and C/N ratios were evaluated in shake-flask experiments under the cultivation conditions described above. Biomass concentration was determined gravimetrically as dry cell weight after drying samples at 105 °C to constant weight. Protein content was determined using the Novagen™ BCA Protein Assay Kit according to the manufacturer's instructions [13], following the cell lysis protocol of Guerlava et al. [14], modified by NaOH-SDS extraction and heat treatment (100 °C, 5 min) prior to BCA analysis. Amino acid composition was determined after hydrolysis of dried biomass in 6 M HCl/phenol (110 °C, 24 h). Methionine and cysteine were determined after performic acid pre-oxidation following the principles of Commission Regulation (EC) No. 152/2009 [15]. Amino acids were subsequently separated and quantified using a Shimadzu Nexera HPLC system with automated OPA/FMOC pre-column derivatization and fluorescence detection following published methods [16,17]. The dataset includes amino acid profiles (essential and non-essential amino acids, expressed as g/100 g pure protein), enabling independent assessment of protein quality and calculation of amino acid quality indices, including the amino acid score (AAS) and essential amino acid index (EAAI).

### 4.3 Mutagenesis and lethality assessment

Random mutagenesis was performed using EMS following methods described in previous studies [[18], [19], [20], [21], [22]], with minor modifications, including the EMS concentration and neutralization procedure. Cells were harvested, washed twice with sterile 50 mM potassium phosphate buffer (pH 7.0), and resuspended in the same buffer containing 1–5% EMS. After incubation for 2 h under agitation, the mutagen was neutralized with 5% (w/v) sodium thiosulfate. Treated cells were washed, serially diluted, and plated on agar to determine CFU enumeration. Survival and lethality rates were calculated relative to untreated controls to determine the optimal EMS concentration for subsequent mutant generation.

### 4.4 Inhibitor-based screening

Growth inhibition experiments were performed in 48-well microplates (0.5 mL working volume), each containing 0.44 mL of culture medium and 0.06 mL of inhibitor solution. Three amino acid biosynthesis inhibitors were evaluated: GA, AEC, and MSO. Inhibitor concentrations ranging from 0.5 to 400 mM for GA, 0.5 to 300 mM for MSO, and 0.5 to 499 mM for AEC were tested to identify the minimum concentration that completely inhibited growth of the wild-type strain. Growth was monitored by measuring optical density (OD600) using a microplate reader, and final cell concentrations were verified by hemocytometer counting. Based on the inhibition assay, 0.4 M GA was selected for subsequent mutant screening because it completely inhibited wild-type growth [19].

### 4.5 Mutant screening and evaluation

*C. utilis* cells mutagenized with 4% EMS were adjusted to approximately 1 × 10^6^ cells/mL and inoculated into 48-well microplates containing selective medium supplemented with 0.4 M GA inhibitor. Control conditions included the wild-type strain cultivated in medium with and without GA, as well as mutagenized cells cultivated without the inhibitor. Microplates were incubated under the cultivation conditions described above, and growth was monitored by optical density measurements, microscopic examination, and hemocytometer cell counting. Following the initial incubation (∼162 h), all cultures were transferred to fresh GA-containing medium for a second selection cycle, after which wells showing viable cell proliferation were identified by microscopy. These candidates were subjected to a third serial subcultivation in fresh selective medium to confirm phenotype stability. After the final selection cycle, the best-performing mutants were transferred to shake-flask cultures without inhibitor for further characterization [19]. Biomass concentration, protein content, and amino acid composition were determined as described above.

### 4.6 RSM optimization

Medium optimization was performed using RSM to evaluate the effects of molasses concentration and (NH_4_)_2_SO_4_ concentration on biomass and protein production. A two-factor, three-level central composite design (CCD) was applied in two sequential experiments. In the first RSM experiment, molasses and (NH_4_)_2_SO_4_ concentrations ranged from 80 to 120 g/L and 5–20 g/L, respectively, while in the second experiment the design space was expanded to 100–180 g/L molasses and 1–15 g/L (NH_4_)_2_SO_4_. Each design consisted of 13 experimental runs, including four factorial points, four axial points, and five replicated center points to estimate experimental error and assess model adequacy. Biomass concentration (g/L) and protein yield (g/L) were used as response variables, and second-order polynomial models were fitted to evaluate the effects of the individual factors and their interactions.

### 4.7 Antifoam and bioreactor experiments

Antifoam performance was evaluated in shake-flask experiments using the optimized cultivation medium. Three commercial antifoam agents (J673A, SB2121, and Antifoam C) were tested at 0.1%, 0.5%, and 1.0% (w/v) and compared with a control without antifoam. Biomass production was monitored by dry cell weight to identify an antifoam suitable for bioreactor cultivation without inhibiting yeast growth. Batch cultivation was performed in a 5 L stirred-tank bioreactor with a 4 L working volume using the optimized MAM medium. The cultivation temperature was maintained at 30 °C, pH at 5.0, and dissolved oxygen was controlled at 30% by automatically adjusting agitation (300–800 rpm), aeration (2.5–6.0 slpm), and oxygen supplementation (0–1.5 slpm). Antifoam C (0.5%, w/v) was added to the fermentation medium before inoculation, while an additional 1% Antifoam C solution was connected to the automatic antifoam dosing system for foam control during cultivation. Samples were collected at 0, 16, 25, 40, 51, and 69 h for determination of biomass concentration, cell concentration, protein content, protein yield, and residual sucrose.

### 4.8 Data processing

Unless otherwise stated, all experiments were performed in biological triplicate, and the results are presented as mean ± standard deviation. RSM data were analyzed using Stat-Ease 360 software, version 23.1.8 (64-bit) (Stat-Ease Inc., Minneapolis, MN, USA). Second-order polynomial regression models were fitted to evaluate the effects of the independent variables and their interactions. Model adequacy was assessed using analysis of variance (ANOVA), coefficients of determination (R²), and lack-of-fit tests. The complete experimental datasets, including raw measurements, replicate values, and model outputs, are available in the accompanying Zenodo repository.

## Limitations

The dataset has several limitations related to data acquisition and completeness.

Optical density measurements in microplate experiments showed reduced sensitivity at low cell concentrations, which limited accurate detection of weak growth during inhibitor screening. Additional cell counting was required in some cases, increasing experimental effort.

The dataset includes a limited number of selected mutant candidates after screening, which reflects the applied selection workflow. In addition, amino acid composition data are not available for all samples, and certain amino acids (e.g., tryptophan) were not determined.

Some variability between replicate measurements was observed due to biological and experimental factors. All datasets include standard deviations to reflect this variability.

## Ethics Statement

Authors have read and followed the ethical requirements for publication in Data in Brief. This work does not involve human subjects, animal experiments, or any data collected from social media platforms.

## CRediT Author Statement

**Jelizaveta Palcevska:** Writing - Original draft, Writing - Review & Editing, Investigation; **Zane Kusnere:** Writing - Review & Editing, Methodology; **Svetlana Raita:** Methodology, Writing - Review & Editing, Investigation; **Zane Geiba:** Investigation, Visualizations; **Ilze Vamza:** Data curation, Supervision.

## Data Availability

ZenodoExperimental dataset on mutant generation, amino acid inhibitor-based screening, and process optimization for enhanced protein and essential amino acid production in Candida utilis (Original data).
